# Inhibition of the Striatal Specific Phosphodiesterase PDE10A Ameliorates Striatal and Cortical Pathology in R6/2 Mouse Model of Huntington's Disease

**DOI:** 10.1371/journal.pone.0013417

**Published:** 2010-10-15

**Authors:** Carmela Giampà, Daunia Laurenti, Serenella Anzilotti, Giorgio Bernardi, Frank S. Menniti, Francesca Romana Fusco

**Affiliations:** 1 Santa Lucia Foundation IRCCS Hospital at the European Center for Brain Research, Laboratory of Neuroanatomy, Rome, Italy; 2 Department of Neuroscience, University of Rome Tor Vergata, Rome, Italy; 3 Mnemosyne Pharmaceuticals, Inc., Providence, Rhode Island, United States of America; University of Cambridge, United Kingdom

## Abstract

**Background:**

Huntington's disease is a devastating neurodegenerative condition for which there is no therapy to slow disease progression. The particular vulnerability of striatal medium spiny neurons to Huntington's pathology is hypothesized to result from transcriptional dysregulation within the cAMP and CREB signaling cascades in these neurons. To test this hypothesis, and a potential therapeutic approach, we investigated whether inhibition of the striatal-specific cyclic nucleotide phosphodiesterase PDE10A would alleviate neurological deficits and brain pathology in a highly utilized model system, the R6/2 mouse.

**Methodology/Principal Findings:**

R6/2 mice were treated with the highly selective PDE10A inhibitor TP-10 from 4 weeks of age until euthanasia. TP-10 treatment significantly reduced and delayed the development of the hind paw clasping response during tail suspension, deficits in rotarod performance, and decrease in locomotor activity in an open field. Treatment prolonged time to loss of righting reflex. These effects of PDE10A inhibition on neurological function were reflected in a significant amelioration in brain pathology, including reduction in striatal and cortical cell loss, the formation of striatal neuronal intranuclear inclusions, and the degree of microglial activation that occurs in response to the mutant huntingtin-induced brain damage. Striatal and cortical levels of phosphorylated CREB and BDNF were significantly elevated.

**Conclusions/Significance:**

Our findings provide experimental support for targeting the cAMP and CREB signaling pathways and more broadly transcriptional dysregulation as a therapeutic approach to Huntington's disease. It is noteworthy that PDE10A inhibition in the R6/2 mice reduces striatal pathology, consistent with the localization of the enzyme in medium spiny neurons, and also cortical pathology and the formation of neuronal nuclear inclusions. These latter findings suggest that striatal pathology may be a primary driver of these secondary pathological events. More significantly, our studies point directly to an accessible new therapeutic approach to slow Huntington's disease progression, namely, PDE10A inhibition. There is considerable activity throughout the pharmaceutical industry to develop PDE10A inhibitors for the treatment of basal ganglia disorders. The present results strongly support the investigation of PDE10A inhibitors as a much needed new treatment approach to Huntington's disease.

## Introduction

Huntington's disease (HD) is a devastating neurodegenerative condition characterized by progressive and severe cognitive, emotional, and motor dysfunction, and premature death [1]. The disease is caused by expansion of a CAG repeat in exon 1 of *IT15*, which encodes for the protein huntingtin [2]. The expanded n-terminal poly-glutamine tract in mutant huntingtin is the toxic species [3], with the length of the expansion correlating with age of onset and, to a lesser extent, disease course [4]. Although huntingtin is widely expressed, HD is associated with a specific pattern of neurodegeneration. The striatal medium spiny neurons are most susceptible and loss of striatal volume is the prominent pathological finding [5]. However, cortical pathology is also evident and contributes to the overall dramatic loss of brain volume (up to 40%) in late stage disease [6]. At present, there are few options to treat the symptoms of HD and no therapy to slow the neurodegenerative process.

Mechanisms underlying the selective neuronal vulnerability to mutant huntingtin are under intense investigation but are not fully understood. Aggregates of mutant huntingtin are found in various neuronal compartments in autopsy samples of HD brain [7] and such aggregates are recapitulated in cell culture and transgenic mouse models [8]. However, the role of these aggregates in the neurotoxic process is controversial [9]. The effects in experimental systems of the expression of mutant huntingtin, or a poly-glutamine n-terminal fragment, are diverse [10]. Findings from such studies have suggested a number of possible cause/effect relationships in the neurotoxic process and, importantly, have formed the basis for developing therapeutic strategies [11]. The strategy of interest here emerges from evidence that mutant huntingtin impairs cAMP signaling [12] and gene transcription mediated by the cAMP response element–binding protein (CREB) [13], [14]. Inhibition of CREB-mediated transcription has been hypothesized to contribute to neuronal loss in HD [15], [16], [17], [18] and a decreased transcription of CREB-regulated genes is observed in HD transgenic animals [16], [19], [20]. These findings suggest that counteracting the decreased cAMP signaling and loss of CREB-regulated transcription may be beneficial in treating HD.

We first interrogated the above therapeutic strategy by targeting the cyclic nucleotide phosphodiesterase type IV (PDE4). PDE4 [21] is one of the eleven families of phosphodiesterases that regulate through metabolic inactivation cyclic nucleotide signaling throughout the body [22], including in brain [23]. PDE4 inhibition results in activation of cAMP signaling pathways and, in particular, increases CREB phosphorylation and activation [24], [25]. We have shown that the PDE4 inhibitor rolipram reduces striatal degeneration in a rat quinolinic acid [26] and in the R6/2 transgenic mouse [27] models of HD. While these results provided support for targeting cAMP and CREB signaling through PDE inhibition, they do not provide a direct path to therapy, since PDE4 inhibitors suffer from severe side effect liabilities that have precluded successful clinical deployment. Thus, we turned to the investigation of another phosphodiesterase, PDE10A, as a more promising target.

PDE10A is highly expressed in the striatal medium spiny neurons [28], [29], [30], where it regulates both cAMP and cGMP signaling cascades [31], [32]. Notably, inhibition of PDE10A with the highly specific inhibitor TP-10 results in robust increase in cAMP and in CREB phosphorylation in striatum [33]. We have previously reported that TP-10 treatment reduces loss of striatal and cortical neurons caused by intrastriatal quinolinic acid injection in parallel to an increase in striatal and cortical CREB phosphorylation [34]. Here we report that TP-10 ameliorates the behavioral and neuropathological sequel of mutant huntingtin expression in the well-established transgenic mouse model of HD, the R6/2 mice.

## Results

### TP-10 drug levels

**Figure 1:**
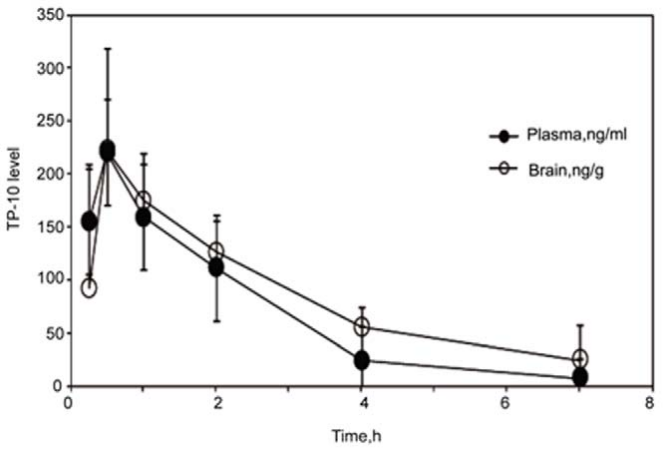
Plasma and brain levels of TP-10 in C57BL/6 mice. Mice were administered TP-10 at 1.5 mg/kg, i.p. and sacrificed at the indicated time to obtain samples of plasma and brain for determination of total TP-10 levels. Plasma levels are presented as ng TP-10/ml and brain levels are presented as ng TP-10/g tissue wet weight. Each data point represents the mean from n = 3 animals. The horizontal dashed line is the total plasma level that, when corrected for plasma protein binding, represents the concentration of TP-10 free in plasma at the IC_50_ of TP-10 for inhibition of PDE10A in vitro (0.3 nM).

TP-10 was administered once daily at a dose of 1.5 mg/kg, i.p. beginning at 4 weeks of age until death. Plasma levels of TP-10, measured in a satellite group of animals, reach a peak within approximately 0.5 h after administration (Figure 1). Brain levels closely tracked the plasma levels and the brain/plasma concentration ratio was 1.3 based on areas under the curve. These pharmacokinetic parameters are very similar to those reported previously for TP-10 administered to mice by the subcutaneous route [33]. Free plasma levels (calculated from the measured total plasma levels corrected for protein binding) reached a maximum of 1.9 nM and remained above the IC_50_ of TP-10 for inhibition of PDE10A in vitro (0.3 nM) for approximately 4 h (Figure 1). Such levels of TP-10 are associated with robust increases in striatal levels of cAMP, cGMP, and CREB phosphorylation and robust efficacy in mouse behavioral models predictive of antipsychotic efficacy [33].

### Loss of righting reflex and weight

**Figure 2:**
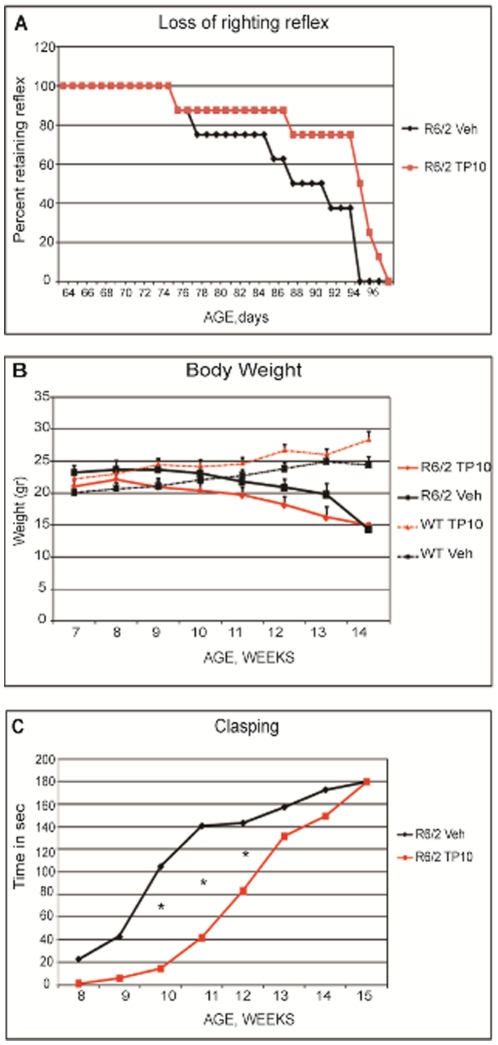
Survival, weight, and clasping response in R6/2 mice treated with TP-10 or vehicle. (**A**) Kaplan-Meyer analysis of survival. R6/2 mice treated with TP-10 had a mean survival time that was significantly (p<0.0001) longer than that of R6/2 mice treated with vehicle. (**B**) Weight as a function of age in the wild type and R6/2 mice. The wild type mice gained weight throughout the course of the study, whereas the R6/2 mice progressively lost weight. There was no statistically significant effect of TP-10 treatment in either genotype. (**C**) The development of the hind-limb clasping phenotype in R6/2 mice treated with TP-10 or vehicle. Two way analysis of variance indicated a statistically significant effect of treatment (F(1,12) = 8,86, p<0.0116) and age (F(6,72) = 25,32, p<0.0000). Post hoc analysis indicated that the TP-10 treated mice showed significantly less clasping at weeks 10, 11, and 12 (*, p<0.01).

Inhibition of PDE10A increased the age at which R6/2 mice lost the righting reflex and were euthanized (Figure 2A). The first R6/2 mouse losing the right reflex was age 76 days. In the R6/2 mice treated with vehicle, 50% of the mice lost the righting reflex by age 88 days and all animals lost the righting reflex by age 95 days. This time course is similar to that observed by others [3] and is consistent with our previous results [27]. The time to loss of righting reflex for R6/2 mice treated with the PDE10A inhibitor TP-10 was significantly extended (p<0.0001) based on a Kaplan-Meier analysis. This difference in the Kaplan-Meier survival curve can best be described by a difference in the rate at which animals lost the righting reflex. In the vehicle treated group, mice lost the righting reflex over 76 to 95 days of age. In contrast, more than 75% of the TP-10 treated mice maintained the ability to right themselves to age 94 days, but then rapidly lost this ability thereafter and all were euthanized over the next week.

Beginning at approximately 10 weeks of age, R6/2 mice exhibited significant weight loss and by 14 weeks the vehicle-treated R6/2 mice were approximately half the size of wild-type littermates. However, TP-10 treatment did not significantly modify the weight loss of the R6/2 mice (Figure 2B).

### Neurological and behavioral assessments

R6/2 mice exhibit an abnormal hind-limb clasping response when suspended by the tail [3] that has been associated with the progression of brain damage [35]–[37]. PDE10A inhibition slowed the development of this neurological abnormality. In vehicle-treated R6/2 mice, the clasping response was evident by 8 weeks of age and reached near maximal levels by 11 weeks (Figure 2C). In contrast, in R6/2 mice treated with TP-10 the clasping response was not evident until 10 weeks of age and then developed more gradually. The time spent exhibiting the clasping response was statistically significantly less in the TP-10 treated mice at weeks 10–12 (Figure 2C).

**Figure 3:**
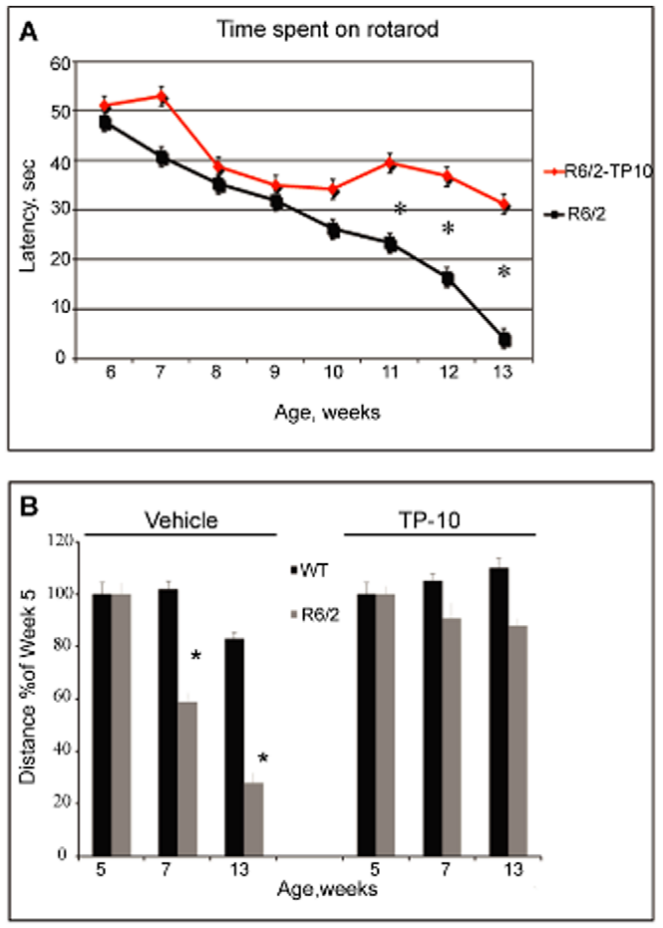
The effect of TP-10 treatment on motor behaviors. (**A**) Latency to fall from the accelerating rotarod in the R6/2 mice. A two way ANOVA indicated an overall significant effect of treatment (F(1,16) = 398,54; p<0.0000) and age (F(7,112) = 149,16; p<0. 0000) and a age X treatment interaction (F(7,112) = 27,4; p<0.0000). R6/2 mice treated with vehicle exhibited a progressive decrease in the latency to fall and this decrease was blunted by TP-10 treatment. Specifically, the TP-10 treated groups had a significantly longer latency to fall compared to the vehicle treated group at weeks 10–12 of age (*, p<0.0002). (**B**) Distance traveled in the open field at ages 5, 7 and 13 weeks. Total distance traveled during a 10 min test session is expressed as a percentage of the distance traveled at 5 weeks for each group (see Table 1 for data from which these percentages were calculated). Wild type mice treated with vehicle exhibited only a slight decrease in distance traveled over the repeated exposures to the open field. In contrast R6/2 mice treated with vehicle exhibited a progressive decrease in distance traveled. This difference between wild type and R6/2 mice was largely ameliorated by TP-10 treatment. The TP-10 treated R6/2 mice had a lesser decrease in distance traveled with repeated testing, as compared to the vehicle treated R6/2 mice. There was a trend for the TP-10 treated wild type mice to travel a slightly greater distance over repeated testing.

Motor coordination was assessed as the ability of mice to maintain balance on an accelerating rotarod at 6 through 13 weeks of age. The wild type mice were able to maintain balance for the duration of each test regardless of age and treatment and TP-10 treatment did not disrupt performance. Because of this ceiling effect, we exclude the data from the wild type mice from our parametric statistical analysis, which is focused exclusively on the two treatment groups of R6/2 mice. As can be seen in Figure 3A, the R6/2 mice became progressively impaired in motor coordination. There was no difference between R6/2 treatment groups during rotarod training and no statistically significant difference initially. Significantly, TP-10 treatment blunted the decline in rotarod performance in the R6/2 mice at later time points. Performance of the vehicle treated R6/2 mice progressed from a slight impairment at 6 weeks of age to complete loss of the ability to maintain balance by age 13 weeks. In contrast, performance deteriorated at a slower rate with TP-10 treatment, as reflected in significantly longer durations in the R6/2 treated compared to vehicle treated animals at weeks 11–13. In fact, at 13 weeks, the TP-10 treated R6/2 mice maintained the ability to balance on the rotarod for an average of 31±6 sec.

**Table 1:** Distance traveled in an open field.

	Vehicle		TP-10	
	WT	R6/2	WT	R6/2
5 weeks	5562±252	4832±217	3370±184	3055±137
7 weeks	5720±172	2863±183	3549±163	2790±28
13 weeks	4650±121	1349±218	3728±225	2689±144

Groups of wild type (WT) or R6/2 mice treated with vehicle or TP-10 were placed in a circular open field at ages 5, 7, and 13 weeks and the total distance traveled (cm) over 10 min was recorded. Each value is the mean ± S.E.M. The statistical analysis indicated an overall significant effect of genotype (F(1,59) = 42,20;P<0,0000) and treatment effect (F(1,59) = 22,74; P<0,0000) and a genotype X treatment interaction (F(1,59) = 20,78; P<0,000) and lastly a treatment X age interaction (F (2,59) = 8,04; P<0,0008. Post hoc analysis indicated that wild type mice traveled a significantly greater distance than the R6/2 mice (P<0,0004). R6/2 mice treated with TP-10 traversed a significantly greater distance than did vehicle treated R6/2 mice (P<0.017). These data, expressed as a percentage distance traveled at week 5, are also presented in Figure 3.

Locomotor activity in an open field was assessed at ages 5, 7 and 13 weeks. Total distance traveled and speed in the open field were recorded; however, since the differences between genotypes and the effect of TP-10 treatment were identical for these two measures, only the data for distance traveled is presented for simplicity (Table 1). The initial statistical analysis included genotype, treatment, and age as main factors in a three way ANOVA. There was a significant main effect of treatment, reflective of the fact that TP-10 treatment reduced distance traveled in the open field in both the wild type and R6/2 mice (Table 1). This is consistent with previous findings for PDE10A inhibition on this measure of locomotor/exploratory behavior [33]. The salient observation in the present study is that PDE10A inhibition with TP-10 blunted the decline in locomotor activity in the R6/2 mice as a function of age (Figure 3B). In the vehicle treated wild type mice, there was only a slight (∼14%) decline in distance traveled from age 5 weeks to age 13 weeks, whereas in the vehicle treated R6/2 mice, distance traveled declined from the level at 5 weeks to a greater extent at 7 (∼40%) and 13 (∼70%) weeks. With TP-10 treatment, the wild type group evidenced a small (∼10%) increase in distance traveled with increasing age. Significantly, distance traveled declined only slightly as a function of age in the R6/2 mice treated with TP-10. In fact, there was only a 12% decline in the distance traveled between 5 weeks and 13 weeks of age in this group, and the difference in distance traveled at 13 weeks was only 18% lower in the R6/2 mice as compared to the wild type mice.

### Neuropathological outcome measures

A cohort of R6/2 mice treated with TP-10 or saline, and a comparison group of wild type mice treated with vehicle, were sacrificed at 13 weeks of age and brains were processed for analysis of mutant huntingtin-induced neuropathology.

**Figure 4:**
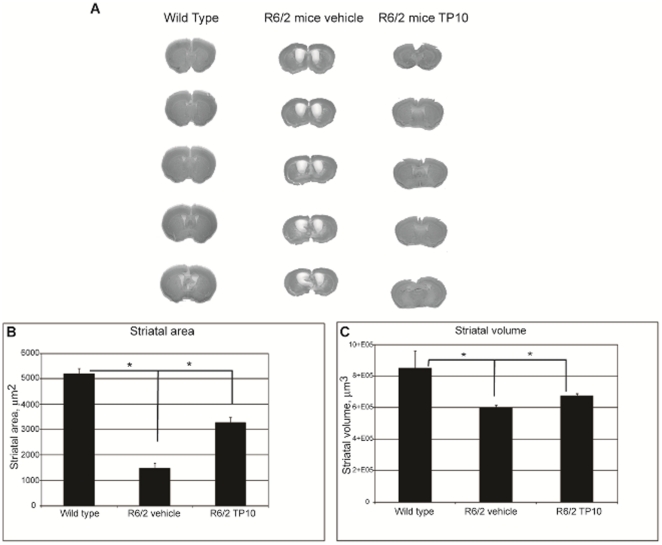
Effects of TP-10 treatment on striatal atrophy in R6/2 mice at 13 weeks of age. (**A**) Transmitted light microscope images showing representative Nissl-stained coronal sections of a wild-type mouse, a vehicle treated R6/2 mouse and a TP10-treated R6/2 mouse, (left to right, respectively). Marked gross atrophy and enlarged lateral ventricles are present in the sections from the vehicle treated R6/2 mouse compared the wild type mouse. These differences are largely absence from the sections of the R6/2 mouse treated with TP-10 from 4 to 13 weeks of age. (**B**) Quantification of differences in striatal area. Striatal area was calculated (see Methods) from sections taken from wild type and vehicle- or TP-10 treated R6/2 mice (n = 6/group). An one-ANOVA indicated an overall significant effect of group (F (2,24) = 62.66; p<0.0000). Post hoc analysis indicated that R6/2 mice treated with vehicle had a significantly reduced striatal area compared to the wild type group. The striatal area of R6/2 mice treated with TP-10 was significantly greater than that of the vehicle treated R6/2 mice (p<0.00014), yet significantly smaller than that of the wild type mice (p<0.00013). (**C**) Quantification of differences in striatal volume in the same groups as (B). An ANOVA indicated an overall significant effect of group (F (2,5) = 7,14; p<0.03). Post hoc analysis indicated that R6/2 mice treated with vehicle had a significantly reduced striatal volume compared to the wild type group. The striatal volume of R6/2 animals treated with TP-10 was intermediate between these two groups, and was significantly greater than that of the vehicle treated R6/2 mice (p<0.03), yet significantly smaller than that of the wildtype mice (p<0.03).

Expression of mutant huntingtin has pervasive effects on different aspects of brain morphology. At the grossest level, this is reflected in an overall reduction in brain volume. The area of brain most susceptible to mutant huntingtin-induced pathology is striatum and we examined the effect of PDE10A inhibition on this pathology in detail. In agreement with previous reports [38], [39], the size of the striatum was markedly reduced in R6/2 mice compared to wild-type littermates, as illustrated in coronal sections at representative planes in Figure 4A. The area of striatal tissue in the step series of coronal sections was first determined. The striatal area of vehicle treated R6/2 mice (1.4±0.2×10^5^ µm^2^) was only 27% of that of the wild-type animals (5.2±0.2×10^5^ µm^2^; Figure 4B). Treatment of the R6/2 mice with TP10 statistically significantly reduced by 50% the loss of striatal size (3.3±0.2×10^5^ µm^2^; Figure 4B). The total volume of striatum was reconstructed from the step series of coronal sections, encompassing the full rostrocaudal extent of the striatum. Calculated striatal volume of vehicle-treated R6/2 mice (6.0±0.1×10^5^ µm^3^) was approximately 70% that of wild type mice (8.5±0.1×10^5^ µm^3^; Figure 4C). This atrophy was partially but significantly ameliorated by treatment of the R6/2 mice with TP10 (striatal volume of 6.7±0.1×10^5^ µm^3^; Figure 4C).

**Figure 5:**
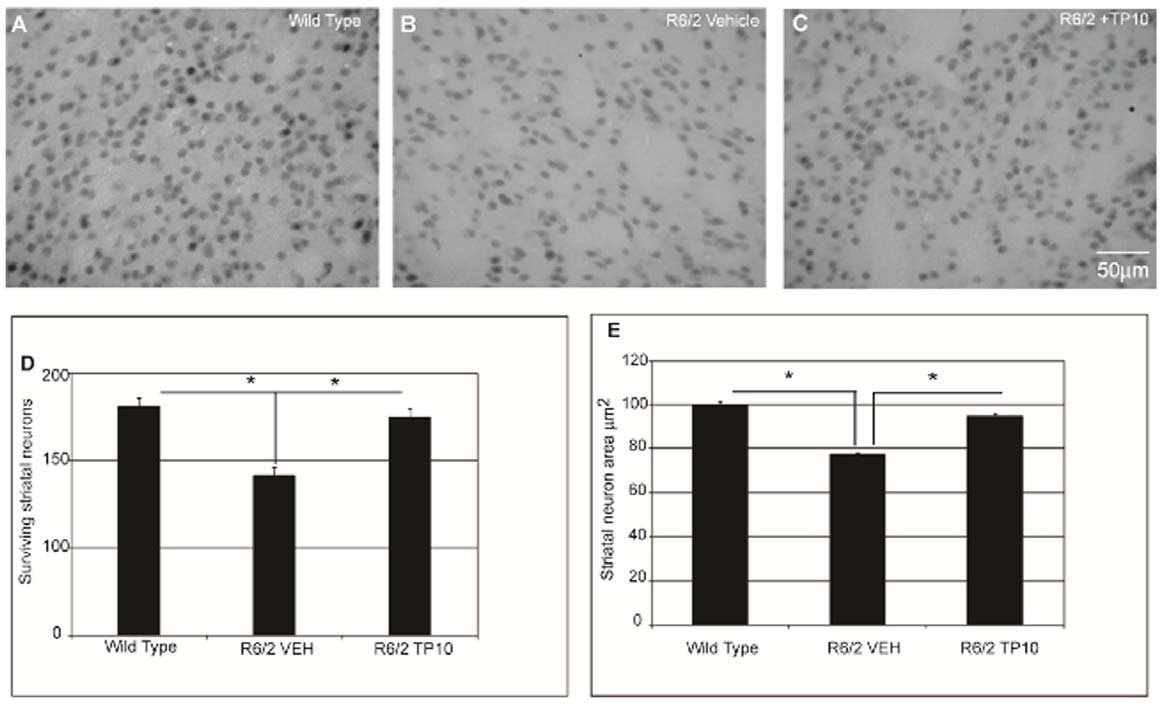
Effects of TP-10 treatment on striatal neuron number and volume in R6/2 mice. Representative photomicrographs of DAB immunohistochemistry for the marker of the medium spiny neurons, calbindin-28K (CALB, black), in the striatum of a (**A**) wild-type mouse, (**B**) vehicle treated R6/2 mouse, and (**C**) R6/2 mouse treated with TP10 from 4 to 13 weeks of age. There is a marked decrease in neuronal count in the sample from a vehicle treated R6/2 mice compared to wild-type mice and TP10 treated R6/2 mice. (**D**) Quantification of the number of striatal neurons labeled with CALB. Striatal neuron counts were carried out (see Methods) on sections from wild type and vehicle- or TP-10-treated R6/2 mice (n = 6/group). An ANOVA indicated an overall significant effect of group (F(2,90) = 36,48; p<0.0000). Post hoc analysis indicated that R6/2 mice treated with vehicle had a significantly reduced density of CALB-positive neurons in striatum compared to the wild type group. The density of striatal neurons in R6/2 mice treated with TP-10 was not statistically different to that of wild type mice, and was significantly greater than that of the vehicle treated R6/2 animals (p<0.0001). (**E**) Area of striatal neuron cell bodies labeled with CALB. Cell body area of approximately 400 neurons per animal (n = 6/group) was measured using Zeiss LSM software. An ANOVA indicated an overall significant effect of group (F(2,899) = 61,64; p<0.0000). Post hoc analysis indicated that R6/2 mice treated with vehicle had a significantly reduction in the size of striatal neurons compared to wild type animals (p<0.00002). The size of striatal neurons in R6/2 animals treated with TP-10 was not statistically different to that in wild type animals, and was significantly greater than that of the vehicle treated R6/2 animals (p<0.00002).

Striatal pathology in the R6/2 mice was also reflected in a reduction in the number of CALB-positive neurons (Figure 5). In the wild-type mice, the average number of striatal neurons per mm^2^ was 180±5, and was statistically significantly reduced to 141±4 neurons in the vehicle-treated R6/2 mice (Figure 5A compared to 5B). PDE10A inhibition nearly completely prevented the decrease in neuron number, with 175±4 NeuN positive neurons found per field in the TP10-treated R6/2 mice (Figure 5C). ANOVA indicated that there was a statistically significant difference in striatal neuron number. Post hoc analyses indicated that the vehicle-treated R6/2 mice had a significantly lower number of striatal neurons than did either the wild type mice or the TP-10 treated R6/2 mice; the latter two groups did not differ (Figure 5D).

A more detailed examination of the effect of TP-10 treatment was carried out in sections stained for calbindin 28K (CALB), which is a striatal- specific marker of the medium spiny neurons. Visual observation revealed that the CALB-positive neurons of vehicle treated R6/2 mice exhibited a previously described shrunken appearance [3] and were of apparently smaller size than in wild-type mice. Quantitative analysis using confocal microscopy confirmed that the average area of CALB positive neurons in the R6/2 mice (77.2±1.1 µm^2^) was indeed statistically significantly smaller than in wild-type mice (99.9±1.3 µm^2^; Figure 5E). Treatment of the R6/2 mice with TP-10 almost completely ameliorated this change in striatal neuron appearance and size (94.8±1.2 µm^2^; Figure 5E).

**Figure 6:**
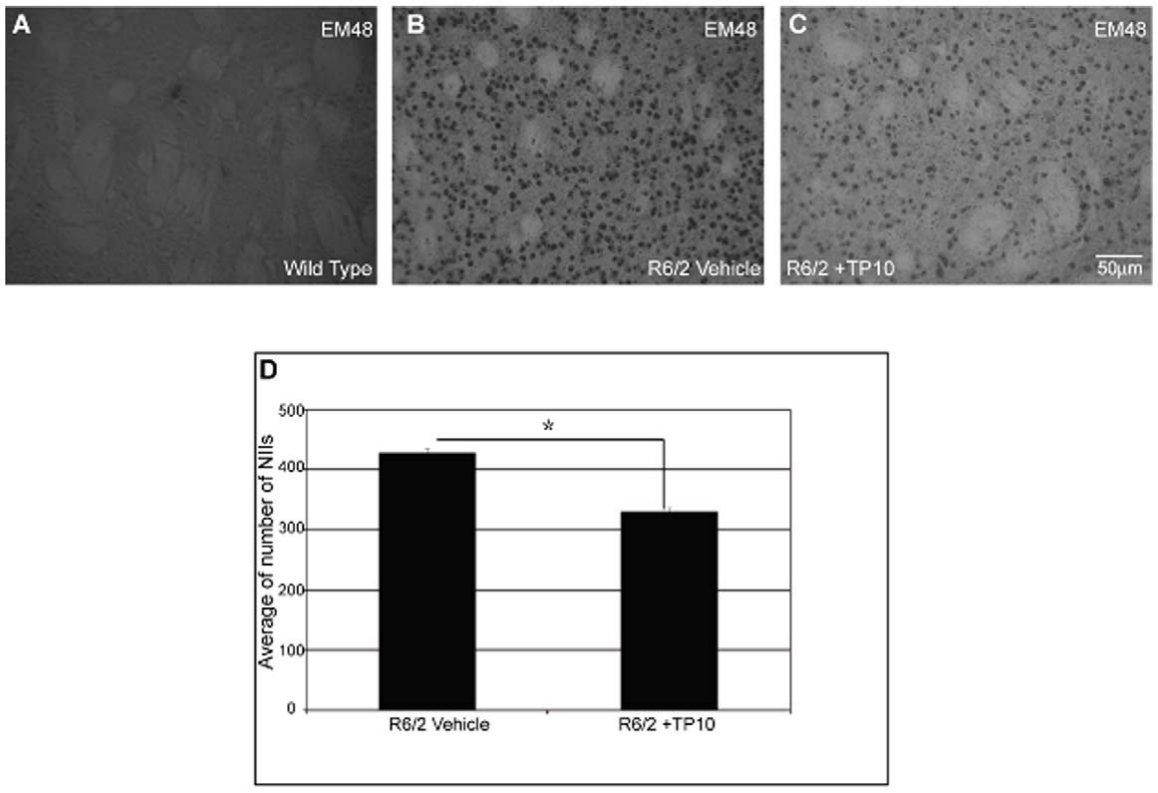
Effects of TP-10 treatment on the density of NIIs in the striatum of R6/2 mice. Representative photomicrographs of DAB immunohistochemistry for EM48 (black), which labels neuronal intranuclear inclusions containing aggregated mutant huntingtin (NIIs) in the striatum of a (**A**) wild type, (**B**) vehicle treated R6/2, or (**C**) R6/2 mouse treated with TP10 from 4 to 13 weeks of age. (**D**) Quatification of NIIs in vehicle- or TP-10 treated R6/2 mice (see methods, n = 6/group). There were no NIIs detected in striatum of wild type mice, so this group was not included in the statistical analysis. A t-test indicated that the density of NIIs in striatum of R6/2 mice treated with TP-10 was lower than that in R6/2 mice treated with vehicle (p<0.0001).

The expression of exon 1 of mutant huntingtin in the R6/2 mice results in the formation of neuronal intranuclear inclusions (NIIs), detected with the antibody EM-48 [40]. EM-48 immunoreactivity was abundant in striatum from the vehicle-treated R6/2 mice (Figure 6). In fact, 428±8 NIIs/mm^2^ were observed in these animals (Figure 6 B). The average number of NIIs was significantly reduced by 25% to 330±5 NIIs/mm^2^ in mice treated with TP-10 (Figure 6C, D). NIIs were not observed in wild type mice Figure 6A).

**Figure 7:**
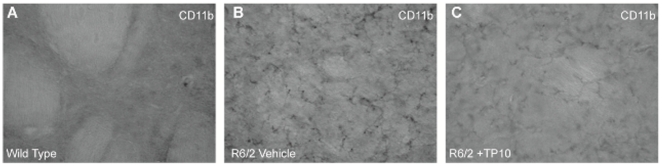
Effects of TP-10 treatment on reactive microglia in the striatum of R6/2 mice. Representative photomicrographs of DAB immunohistochemistry for the marker of activated microglia, CD11b (black), in the striatum of a (**A**) wild type, (**B**) vehicle treated R6/2, or (**C**) R6/2 mouse treated with TP10 from 4 to 13 weeks of age. In the sample from the vehicle treated R6/2 mouse, there is an apparent intense microglial reaction, in which microglial cells appear numerous and with coarse arborizations and a rod-shaped body. In the sample from a TP-10 treated R6/2 mouse, there are fewer reactive cells, along with quiescent cells.

The striatal neuron damage caused by expression of mutant huntingtin induces the activation of microglia (Figure 7). In wild-type mice, only scattered microglia were visualized by CD-11 immunostaining (Figure 7A). These had the ovoid body and lack of cellular processes characteristic of the quiescent state. In contrast, an intense microglial reaction was evident in the vehicle treated R6/2 group, where microglial cells appeared numerous, had rod-shaped cell bodies with coarse arborizations (Figure 7B). This microglial reaction was attenuated in TP-10 treated R6/2 mice, with fewer reactive cells evident among the normal quiescent cells (Figure 7C).

**Figure 8:**
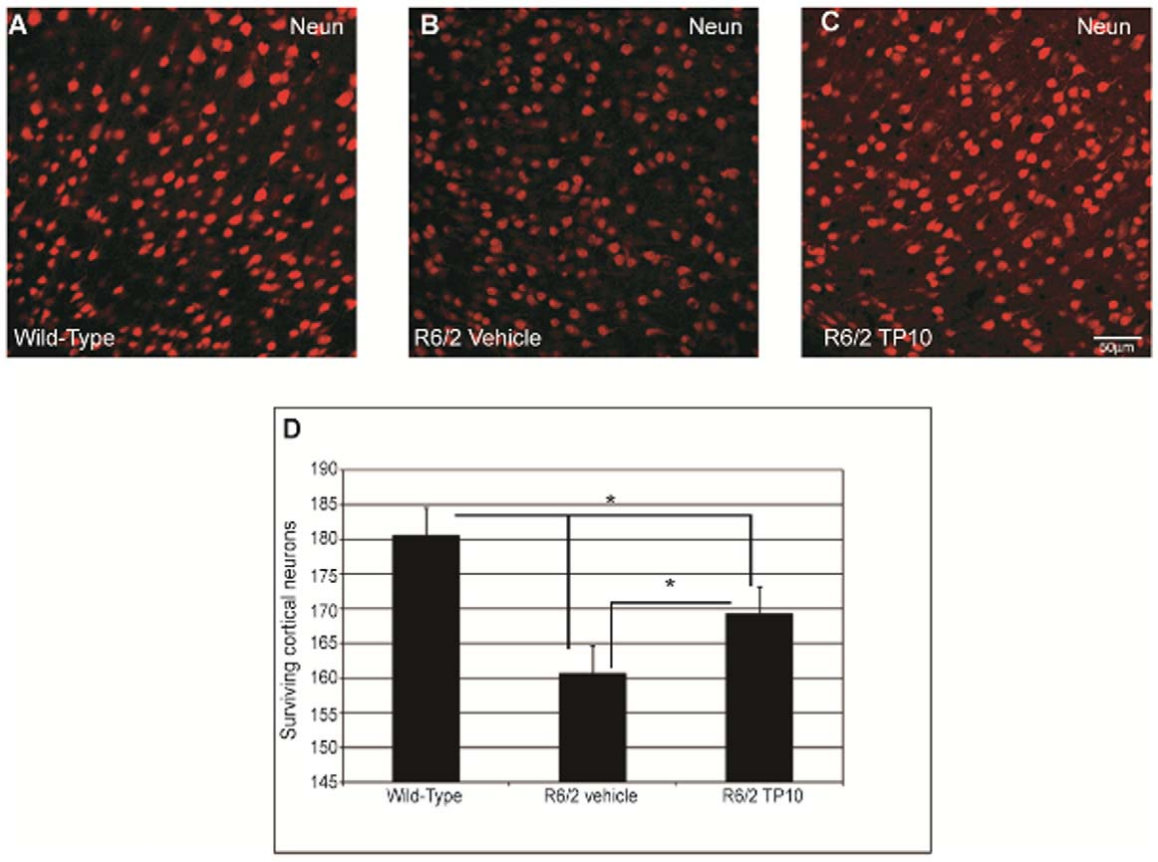
Effects of TP-10 treatment on the density of cortical neurons in R6/2 mice. Representative photomicrographs of single label immunofluorescence for the neuronal marker NeuN (red) to label cortical neurons in samples taken from a (**A**) wild-type, (**B**) a vehicle treated R6/2, or (**C**) R6/2 mouse treated with TP10 from 4 to 13 weeks of age. There is a marked decrease in neuronal count in the sample from a saline treated R6/2 mice compared to wild-type mice and TP10 treated R6/2 mice. (**D**) Quantification of the number of cortical neurons labeled with NeuN. Cortical neuron counts were carried out (see Methods) on sections from wild type and vehicle- or TP-10-treated R6/2 mice (n = 6/group). An ANOVA indicated an overall significant effect of group (F(2,39) = 3,61; p<0.03). Post hoc analysis indicated that R6/2 mice treated with vehicle had a significantly reduced density of NeuN-positive neurons in cortex compared to the wild type group (p<0.04). The density of cortical neurons in R6/2 animals treated with TP-10 was intermediate between these two groups, and was significantly greater than that of the vehicle treated R6/2 mice (p<0.04), yet significantly smaller than that of the wild type mice (p<0.04).

Expression of mutant huntingtin in the R6/2 mice also results in cortical pathology (Figure 8). The number of NeuN positive neurons per mm^2^ was significantly lower in vehicle treated R6/2 mice (160±3) compared to wild type mice (181±7; Figure 8B vs. 8A). PDE10A inhibition partially ameliorated this loss of cortical neurons (Figure 8C). The number of NeuN positive neurons was statistically significantly higher in the TP-10 treated R6/2 mice (169±4) than in vehicle treated R6/2 mice, and was not statistically different from that in the wildtype mice (Figure 8D).

### Immunohistochemical analysis of pCREB and BDNF

PDE10A regulates cAMP signaling and activation of the transcription factor CREB in striatum [33]. Deficiency in these signaling cascades is hypothesized to contribute to the pathology caused by mutant huntingtin in mice and in HD patients. One of the key downstream mediators in this regard is BDNF, a principal neurotrophic factor for both striatal and cortical neurons. Thus, it was of interest to investigate the effects of PDE10A inhibition on CREB phosphorylation (pCREB) and BDNF in the R6/2 model used here.

**Figure 9:**
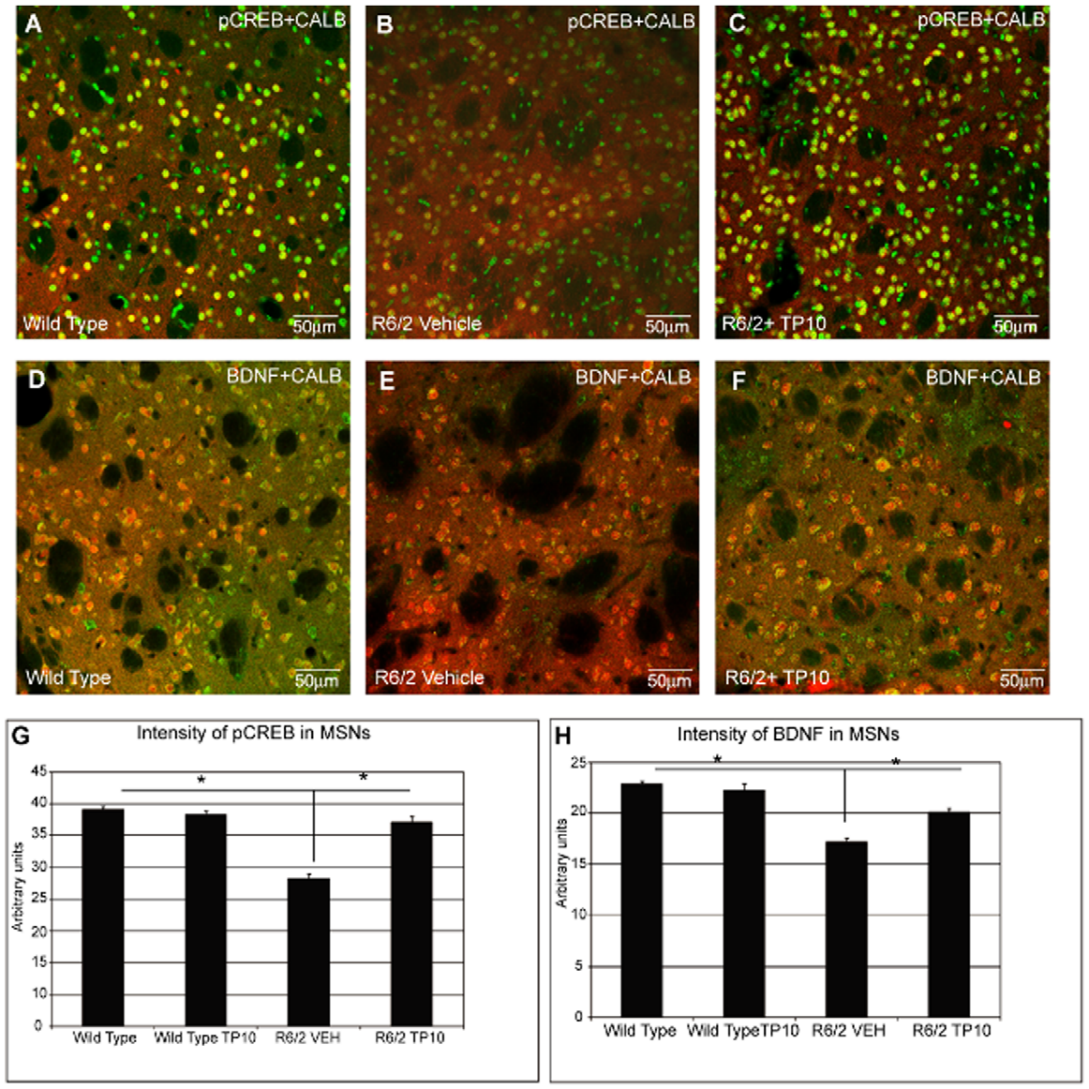
Effects of TP-10 treatment on pCREB and BDNF in the striatum of R6/2 mice. Representative confocal laser scanning microscopy images of dual-label immunofluorescence for CALB (red) and pCREB (green, panels **A–C**) or BDNF (green, panels **D–E**) in striatal samples from a (**A, D**) wild-type, (**B, E**) vehicle treated R6/2, or (**C, F**) R6/2 mouse treated with TP-10 from 4 to 13 weeks of age. For both pCREB and BDNF, there is a reduced density of immunoreactivity in the R6/2 vehicle treated samples relative to the wild type samples. This decrease is largely eliminated in samples from R6/2 mice treated with TP-10. (**G**) Quantification of the intensity of pCREB immunoreactivity associated with CALB-labeled striatal neurons. The intensity of pCREB immunoreactivity associated with CALB-positive cells were carried out (see Methods) on sections from wild type or R6/2 mice treated with vehicle or TP-10 (n = 6/group). A two way ANOVA indicated an overall significant effect of genotype (F(1,1008) = 103,76; p<0.0000) and treatment (F(1,1008) = 50,59; p<0.0000) and a genotype X treatment interaction (F(1,1008) = 50,59; p<0.0000). Post hoc analysis indicated that there was no statistically significant difference in pCREB level in wild type mice treated with TP-10 or vehicle. R6/2 mice treated with vehicle had a significantly reduced pCREB level compared to the vehicle treated wild type group (p<0.00002). The pCREB level in R6/2 animals treated with TP-10 was not statistically different to that in the wild type groups, and was significantly greater than that of the vehicle treated R6/2 animals (p<0.0000). (**H**) Quantification of the intensity of BDNF immunoreactivity associated with CALB-labeled striatal neurons. A two way ANOVA indicated an overall significant effect of genotype (F(1,1270) = 130.23; p<0.0000) and treatment (F(1,1270) = 15.63; p<0.0001) and a genotype X treatment interaction (F(1,1270) = 15.63; p<0.0001). Post hoc analysis indicated that there was no statistically significant difference in BDNF level wild type mice treated with TP-10 or vehicle. R6/2 mice treated with vehicle had a significantly reduced BDNF level compared to the vehicle treated wild type group (p<0.00002). The BDNF level of R6/2 animals treated with TP-10 was not statistically different to that in the wild type groups, and was significantly greater than that of the vehicle treated R6/2 animals (p<0.0000).

Striatal medium spiny neurons were labeled with antibodies to CALB and the intensity of pCREB or BDNF immunoreactivity associated with the surviving CALB-positive neurons was quantified (Figure 9). The intensity of pCREB labeling was statistically significantly lower in the neurons of the vehicle treated R6/2 mice (28.2±0.6 arbitrary units) compared to the wild-type littermates (39.0±0.5; Figure 9B vs. 9A, 10G). This difference was ameliorated by PDE10A inhibition, with pCREB levels in TP10-treated R6/2 mice (37.0±0.9) restored to the same level as in the wild-type mice (Figure 9C, 9G). The level of BDNF immunoreactivity associated with the striatal medium spiny neurons was also reduced in vehicle-treated R6/2 mice (17.1±0.3) compared to wild-type mice (22.8±0.3; Figure 9D vs 9E). TP-10 treatment of the R6/2 mice resulted in statistically significantly higher levels of BDNF (20.1±0.3) compared to vehicle treated R6/2 mice, an increase of 40% (Figure 9F, 9H).

**Figure 10:**
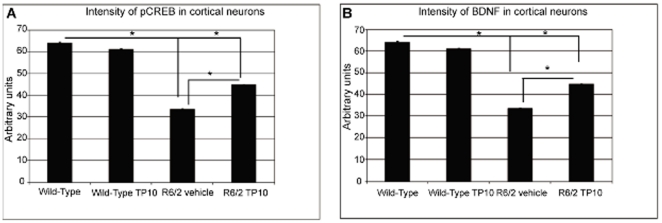
Effect of TP-10 treatment on pCREB and BDNF levels in cortex of R6/2 mice. (**A**) Quantification of the intensity of pCREB immunoreactivity associated with NeuN-labeled cortical neurons at age 13 weeks. The intensity of pCREB immunoreactivity associated with NeuN-positive cells were carried out (see Methods) on sections from wild type or R6/2 mice treated with vehicle or TP-10 from 4 to 13 weeks of age (n = 6/group). A two way ANOVA indicated an overall significant effect of genotype (F(1,1558) = 868.66; p<0.000) and treatment (F(1,1558) = 45.46; p<0.0000) and a genotype X treatment interaction (F(1,1558) = 45.46; p<0.0000). Post hoc analysis indicated that there was no statistically significant difference in pCREB level in wild type mice treated with TP-10 or vehicle. R6/2 mice treated with vehicle had a significantly reduced pCREB level compared to the vehicle treated wild type group. The pCREB level in R6/2 animals treated with TP-10 was not statistically different to that in the wild type groups, and was significantly greater than that of the vehicle treated R6/2 animals (p<0.000). (**B**) Intensity of BDNF immunoreactivity associated with NeuN-labeled cortical neurons. A two way ANOVA indicated an overall significant effect of groups (F(1,1148) = 540.66; p<0.000) and treatment (F(1,1148) = 52.13; p<0.0000) and a genotype X treatment interaction (F(1,1148) = 52.69; p<0.000). Post hoc analysis indicated that there was no statistically significant difference in BDNF level wild type mice treated with TP-10 or vehicle. R6/2 mice treated with vehicle had a significantly reduced BDNF level compared to the vehicle treated wild type group. The BDNF level of R6/2 animals treated with TP-10 was statistically different to that in the wild type groups, and was significantly greater than that of the vehicle treated R6/2 animals (p<0.000008).

In NeuN-positive cortical neurons, the levels of both pCREB and BDNF were statistically significantly reduced in vehicle treated R6/2 mice (40±0.3 vs. 34±0.4 arbitrary units, respectively) compared to wild-type mice (55±0.6 and 64±0.5; Figure 10). TP-10 treatment of the R6/2 mice partially but statistically significantly ameliorated these decreases. Specifically, pCREB levels were 40% higher (46±0.3) and BDNF levels were 30% higher (45±0.3) in the TP-10 treated compared to vehicle treated R6/2 mice.

## Discussion

The CAG expansion in the protein huntingtin that causes HD is selectively detrimental to striatal medium spiny neurons and cortical pyramidal neurons, despite a much broader expression [5], [6]. Studies in experimental systems and findings from post mortem brain tissue from HD patients have revealed a number of effects of mutant huntingtin that may underlie this neurotoxicity [10], [11]. However, it is not clear which of these effects are causal or that account for the selective vulnerability of cortical and striatal neurons. It is also unresolved as to how toxicity in one of these susceptible neuronal populations impacts the vulnerability of the other. Here we report that pharmacological inhibition of the striatal specific phosphodiesterase, PDE10A, ameliorates both striatal and cortical neuropathology in the R6/2 mice. These results have a number of mechanistic implications. These findings also support the use of PDE10A inhibitors as a new therapeutic approach to HD. This is particularly significant since a number of such compounds are in clinical development. These points are elaborated below.

PDE10A is one of the superfamily of phosphodiesterases that regulate through metabolic inactivation cyclic nucleotide signaling throughout the body. PDE10A is expressed at high levels in striatal medium spiny neurons, where it is distributed throughout the soma, dendritic, and axonal compartments [28]–[30]. PDE10A mRNA is also detected broadly throughout the brain, including in cortex [29]. However, mRNA levels are 20-fold or more lower than in striatum, and only very low levels of PDE10A-like immunoreactivity, confined to the nuclear/perinuclear region, are detected with one antibody [28], [29] but not a second [41]. Acute pharmacological inhibition of PDE10A results in a robust increase in cAMP and cGMP levels in striatum in wild-type mice and rats [31], [33]. In mice, the PDE10A inhibitor-induced increase in cAMP results in increased phosphorylation of CREB in striatum [33], as well as the induction of striatal of cfos and neuropeptide mRNA expression [42], [43]. In contrast, there are no detectable changes in any of these biochemical markers in non-striatal tissues [33], [43]. These results indicate that the most significant, if not exclusive, effect of PDE10A inhibition is on cyclic nucleotide signaling in striatal medium spiny neurons.

In the present study, we found that PDE10A inhibition had a significant effect on the development of neurological deficits in the R6/2 mouse, a model of HD [3]. The R6/2 mice express the first exon of the *huntingtin* gene engineered to contain ∼150 CAG repeats. Beginning at ∼8 weeks of life these mice develop a number of behavioral and neuropathological sequelae mirroring pathologies observed in HD patients. Life span is significantly shortened to 12 to 15 weeks [3], [27], with differences in life span observed between laboratories accounted for, at least in part, by differences in animal husbandry. We administered to R6/2 mice the PDE10A inhibitor, TP-10, beginning at 4 weeks of age, before the development of neurological impairment, until the time of euthanasia. We found that PDE10A inhibition significantly delayed the induction of in-life neurological impairment. TP-10 treatment slowed the development of the hind-limb clasping reflex, a neurological abnormality that tracks disease progression [3], [35]–[37], and also reduced deficits in rotarod performance and in open field activity. The age at which R6/2 mice lost the righting reflex and were euthanized was significantly extended by treatment with the PDE10A inhibitor TP-10, based on a Kaplan-Meier analysis. This difference in the Kaplan-Meier curve is best described as a difference in the rate at which the treatment groups lost the righting reflex. Specifically, ∼75% of the TP-10 treated R6/2 mice maintained the ability to right for 13 weeks, after which the animals were rapidly lost. In contrast, animals were lost from the vehicle treated group at a gradual rate beginning at 11 weeks. It is interesting to note that TP-10 treatment did not alter the significant weight loss observed in the R6/2 mice. We speculate that the rapid decline of the TP-10 treated mice at week 14 of age may have been due to this feature of the phenotype that overwhelmed the beneficial effects on neurological function.

Corresponding to these in-life effects, PDE10A inhibition ameliorated neuropathology in the R6/2 mice. TP-10 treatment reduced by 50% the loss of striatal area and nearly completely ameliorated the loss of and morphological changes in the medium spiny neurons, including the quantitative reduction in soma size. Medium spiny neurons in the R6/2 mice accumulate NIIs comprised of aggregates of the poly-glutamine peptide encoded by the *huntingtin* exon 1 transgene [8]. TP-10 treatment significantly reduced the density of these aggregates in striatum. PDE10A inhibition also had a significant beneficial effect on cortical pathology in the R6/2 mice. TP-10 treatment decreased the reduction in cortical neuron counts by 40%. Finally, TP-10 treatment was accompanied by a significant increase in phosphorylated CREB and BDNF in both striatum and cortex.

The beneficial effects of PDE10A inhibition on striatal pathology are readily rationalized. PDE10A is expressed at high levels in medium spiny neurons and inhibiting the enzyme results in a robust increase in striatal cAMP levels and activation of cAMP-mediated signaling pathways [31], [33], [42]. The increase in medium spiny neuron cAMP signaling that results from PDE10A inhibition may be trophic to these neurons via a number of downstream mechanisms [12]. PDE10A inhibition may also ameliorate HD-specific deficits in cAMP signaling, as proposed to occur based on findings in HD models [12] and as evidenced by decreased levels of cAMP in cerebrospinal fluid sampled from HD patients [44]. One of the deficits in cAMP regulated-signaling that has been hypothesized to be specifically involved in the development of HD pathology is reduced transcription mediated by the transcription factor CREB [13]–[20]. PDE10A inhibition in normal mouse brain produces a robust increase in CREB activation downstream of cAMP [31], [33] as well as an increase in transcription of cfos and striatal neuropeptide genes [42], [43]. Furthermore, we observe here a significant increase in phosphorylated CREB in brain of R6/2 mice treated with TP-10. It has also been suggested that a causal factor in MSN pathology in HD stems from deficient trophic support due to a decrease in striatal BDNF [45], [46], [47]. We observed a significant increase in BDNF levels in striatum of R6/2 mice with TP-10 treatment. Thus, PDE10A inhibition-induced increases in striatal cAMP signaling, CREB mediate transcription, and BDNF levels all may contribute to the significant amelioration of striatal pathology resulting from treatment of the R6/2 mice with the PDE10A inhibitor TP-10.

The mechanism(s) whereby PDE10A inhibition increases striatal, and cortical, BDNF levels, and ameliorates cortical pathology are more speculative. BDNF is delivered to striatum by anterograde transport from cortical and, to a lesser extent, midbrain striatal inputs [48]. However, retrograde transport of BDNF from striatum back to cortex is also proposed as a significant element of trophic support to the source cortical neurons [48]. We suggest that the amelioration of striatal pathology by PDE10A inhibition may maintain corticostriatal synaptic interconnectivity, with the secondary effect of maintaining retrograde transport of BDNF to cortex. This, in turn, may reduce cortical neuron pathology. However, it is also possible that there is a direct effect of TP-10 treatment on cortical CREB phosphorylation and BDNF synthesis resulting from inhibition of the nuclear/perinuclear PDE10A present in cortex, although there is no evidence for similar effects in non-diseased rodent brain [43]. Nonetheless, given that this is an important mechanistic question, further investigation is warranted. Insight may be garnered from an analysis of the promoter driving the increase in cortical BDNF levels following PDE10A inhibitor treatment [48]. A preferential increase driven from the CREB-regulated exon III promoter may suggest a direct effect of PDE10A inhibition on cortical neurons.

A notable finding is that PDE10A inhibition reduced the number of NIIs. In this respect, PDE10A inhibition is similar to overexpression of BNDF, in that both treatments reduce both neuropathology and NII accumulation [47]. The role of NIIs in the pathology of HD is controversial [9] and it has yet to be resolved whether these protein aggregates are causal for pathology or are protective by providing a sink to reduce the concentration of soluble toxic mutant huntingtin products. The latter hypothesis is based on finding that some treatments that reduce neuronal pathology are accompanied by an increase in NIIs. The effects of PDE10A inhibition and BDNF overexpression suggest that the formation of NIIs is, at least in part, driven by the underlying neuropathology.

The present results strongly support the targeting of deficient cAMP signaling as a therapeutic strategy to slow disease progression in HD. Given that PDE10A inhibition may affect cAMP signaling exclusively in medium spiny neurons, the present findings provide insight into the interrelationship of striatal and cortical pathology in the disease and indicate that targeting striatal pathology may have a broader impact. Finally, the observation that NII accumulation was reduced by PDE10A inhibition suggests that the formation of these aggregates may be driven by more fundamental pathological processes. We note that these conclusions must be limited at present in that only the R6/2 mouse model was examined and only a limited number the behavioral assays and assessments of pathology were possible. It will be of considerable interest to investigate whether similar beneficial effects of PDE10A inhibition are observed in other preclinical models of Huntington's disease using a broader range of analytical techniques.

Perhaps the greatest significance of the present findings in the mouse model of HD is strong support for the investigation of PDE10A inhibitors as a treatment for the human disease. There is significant progress in the clinical development of PDE10A inhibitors, with a number of pharmaceutical discovery programs underway [49], [50] and with PF-2,545,920 (also referred to as MP-10 [Ref 33], a very close structural analog of TP-10) having advanced to Phase II testing [51]. The primary therapeutic indication for PF-2,545,920 is the treatment of schizophrenia. It is noteworthy that HD patients often suffer severe neuropsychiatric symptoms that share commonality with those suffered by patients with schizophrenia. The pathophysiology underlying these symptoms may similarly stem from striatal dysfunction. Thus, it is reasonable to speculate that PDE10A inhibitors may have a symptomatic benefit against the neuropsychiatric symptoms of HD. These neuropsychiatric symptoms often develop well before a formal diagnosis of HD, which is based on the occurrence of chorea. Thus, PDE10A inhibitors may offer HD patients early symptom relief, with long term therapy slowing disease progression.

## Methods

### Ethics Statement

All studies were conducted in accordance with European Communities Council Directive of 24 November 1986 (86/609/EEC) and approved by the Santa Lucia Foundation Animal Care and Use committee (protocol #45/08).

### Animals and treatments

Breeding pairs of mice to generate the R6/2 animals used in this study were obtained in 2009 from Jackson Laboratories (Bar Harbor, ME, stock number 002810). The colony was expanded by breeding Ovarian Transplanted hemizygote females X B6CBAF1/J males as recommended by Jackson Laboratories. The F1 mice used for experiments were genotyped by PCR assay of DNA obtained from tail tissue. The CAG repeat number is reported by Jackson Laboratories as 160+/− 10, but was not independently confirmed. Mice were identified by a randomly assigned code so that the studies were performed blind as to the genetic identity. Mice were handled under the same conditions by one investigator at the same time of day. All the behavioral and histological data were collected by observers who were blinded to treatment. The mice were housed five in each cage under standard conditions with ad libitum access to food and water. The number of animals used for each study is indicated in Table 2.

**Table 2:** Number of animals.

Genotype	Treatment	Behavior and Survival	Neuropathology	pCREB and BDNF
WT	Vehicle	24	6	6
WT	TP10	24	6	6
R6/2	vehicle	20	6	6
R6/2	TP10	21	6	6

The number of animals used for the different endpoints examined.

Treatments beginning at 4 weeks of age included: R6/2 mice administered TP-10 (1.5 mg/kg once per day) dissolved in saline containing 0.5% DMSO, and 0.5% Emulfor™ (Fluka, New York, NY); R6/2 mice administered vehicle only (1 ml/kg once per day), and wild type mice given TP-10 or vehicle. Treatments were by intraperitoneal (i.p.) injection with solutions prepared freshly daily. TP-10 was synthesized at Pfizer Inc. (Groton, CT).

TP-10 levels in plasma and brain at different time points after i.p. administration were determined in a separate group of C57BL/6 mice (n = 3 per time point). Analytical methods were as previously described [33].

### Loss of righting reflex and weight

Animals were weighed daily starting at the beginning of treatment and the average of weekly weight was calculated. The criterion for euthanasia was loss of righting reflex (30 s cutoff), according to Stack and co-workers [10].

### Assessment of neurological and behavioral function

All behavioral testing was conducted at least 6 h after daily treatment.

Mice of the R6/2 strain exhibit a hind-limb clasping phenotype when suspended by the tail [3]. The clasping phenotype has previously been used to study the disease progression in transgenic HD mice [35], [36] where it is considered a measure of neurological impairment [37]. Mice were suspended by their tail for 180 seconds. The total amount of time spent clasping the hind limb was recorded daily and the average duration over each week was calculated.

Motor coordination and balance was assessed using a five-station mouse rotarod apparatus (Rotarod/RS LSI Letica, Biological Instruments, Varese, Italy). In each station, the rod was 6 cm in length and 3 cm in diameter. Mice were trained at 5 weeks of age to maintain balance with increasing speed up to a constant speed of 16 rpm for three consecutive trials. The test sessions were conducted by one rotarod trial administered twice a week from 6 to 13 weeks. In this session, the speed of rotation was increased from 4 to 16 rpm over 60 s. Mice were given 3 trials on the rod, and the latencies to fall were measured and averaged. For the mice that did not fall, a maximum latency of 60 s was assigned.

Motor activity was measured in an open field consisting of a circular arena (60 cm diameter) with the floor painted white and divided into central and peripheral sectors by drawn black lines. The total area of the two sectors was similar (approximately 1400 cm^2^). The apparatus was contained in a soundproof room illuminated by a red ceiling light (80 W). A video camera above the arena was connected to a video recorder and to a monitor located in an adjacent room. Mice were placed in the arena for 10 minutes during which the distance traveled and the speed in the arena was recorded by means of dedicated software (Noldus Information Technologies, Wageningen, the Netherlands). The data were collected when the mice were pre-symptomatic (5weeks), early symptomatic (7weeks) and fully symptomatic (13weeks).

### Neuropathological assessment

A cohort of 13 week old R6/2 mice treated with TP-10 or vehicle, and a corresponding cohort of wild type mice treated with vehicle were sacrificed and brains processed for neuropathological assessments.

### Tissue processing

Animals at 12 weeks of age were deeply anesthetized and then transcardially perfused with saline solution containing 0.01 ml heparin, followed by 60 ml of 4% paraformaldehyde in saline solution. The brains were removed and postfixed overnight at 4°C in 4% paraformaldehyde in saline solution. Brains were then immersed in a cryoprotection solution of 10% sucrose and 20% glycerol in 0.1 M phosphate buffer (PB) with sodium azide 0.02% for 48 h at 4°C. Brains were frozen and then serially sectioned on a sliding microtome at 40 µm thickness. Sections were mounted on gelatin-coated slides and coverslipped with GEL-MOUNT™. Sections were examined using an epi-illumination fluorescence microscope (Zeiss Axioskop 2). A confocal laser scanning microscopy (Zeiss LSM 510) was subsequently used to acquire all the images for quantification. Controls for specificity of immunohistochemical labeling included omission of the primary antibody and use of preimmune normal mouse and rabbit serum as previously described [26], [52]–[54].

### Evaluation of striatal area and volume

Standard Nissl staining was employed on coronal step serial sections from rostral neostriatum through the level of anterior commissure (interaural 4.66 mm/bregma 0.86 mm to interaural 3.34 mm/bregma −0.46 mm) from brains of 4 animals per group. The striatal area was traced in each section and the striatal area was measured using Neurolucida Stereo Investigator software (Microbrightfield, Cochester, VT, USA). The total volume of the striatum was calculated from these area measurements using the same software.

### Number of surviving striatal neurons

Sections were stained with an antibody against the neuron-specific nuclear antigen NeuN (MAB377X, Chemicon, Temecula, CA). Immunoreactivity was visualized using the diaminobenzidine (DAB)–immunoperoxidase technique as previously described [52]. Cells exhibiting a nuclear DAB signal were counted in each of three 1.0-mm-square confocal microscope fields, in each of three spaced coronal sections through the neostriatum in both hemispheres of 6 mice from each treatment group. The average density of straiatal neurons per mm^2^ in each animal, and then in each group was calculated.

### Striatal neuron area

Sections were stained with an antibody against Calbindin 28K (CALB; Sigma-Aldrich, St. Louis, MO), a 28-KDa protein highly expressed by striatal medium spiny neurons. Immunoreactivity was visualized using the diaminobenzidine (DAB)–immunoperoxidase technique as previously described [52]. To measure the area of medium spiny striatal neurons, images were captured from three 1.0-mm-square confocal microscope fields, in each of three spaced coronal sections in each hemisphere of 6 mice from each group. Area of CALB immunoreactivity was calculated using Zeiss LSM software for approximately 400 neurons per animal. The average area of striatal neurons in each animal, and then in each group, was calculated.

### Evaluation of neuronal intranuclear inclusions (NIIs)

Sections counterstained with Nissl were stained with an antibody, EM48 (MAB5374, Chemicon, Temecula, CA) against the mutant huntingtin protein [55]. Immunoreactivity was visualized using the diaminobenzidine (DAB)–immunoperoxidase technique as previously described [52]. Neurons were examined for the presence of an NII (DAB staining) throughout the entire depth of the neuronal nucleus. A sample of approximately 250 neurons per hemisphere in each of 6 mice per treatment group was analyzed and the percentage of striatal neurons containing NIIs was calculated.

### Microglial morphology

Microglial were immunolabeled with mouse anti-CD-11b (AbD Serotec MorphoSys US Inc, Raleigh, NC) and detected with the diaminobenzidine–immunoperoxidase technique, as previously described [52].

### Phosphorylated CREB in the surviving striatal neurons

Double label immunofluorescence was employed to identify the intensity of phosphorylated CREB (pCREB) in the striatal spiny projection neurons [53]. The intensity of pCREB staining was calculated in each of three 1.0-mm-square confocal microscope fields, in each of three rostrocaudally spaced sections on each hemisphere from 6 mice from each saline, TP10 treated R6/2 mice and wild type littermates.

### Phosphorylated CREB in cortical neurons

Dual label immunofluorescence was also employed to quantify the intensity of pCREB in cortical neurons according to the previously described procedure [26]. Cortical neurons were labeled for the expression of NeuN as described above.

### Striatal level of Brain Derived Neurotrophic Factor (BDNF)

Striatal medium spiny neurons were identified by immunolabeling for CALB as described above. Double label immunofluorescence was employed using an antibody against BDNF (anti-mouse BDNF, Immunological Sciences, Italy) using a previously described immunohistochemical protocol [54]. To evaluate the intensity BDNF immunolabeling per medium spiny neuron, the intensity of fluorescent BDNF immunolabeling in each of three 1.0-mm-square confocal microscope fields was first determined in each of three rostrocaudally spaced sections on each hemisphere of 6 mice from each saline, TP10 treated R6/2 mice and wild type littermates. The intensity of BDNF immunoreactivity per field, expressed in arbitrary units, was calculated by Zeiss LSM software and a mean value was obtained. BDNF immunoreactivity that was not contained in CALB immunoreactive (i.e., interneurons) neurons was calculated.

### Statistical analyses

Survival data were analyzed by means of the product limit method of Kaplan and Meier [56], with p value <0.0001 considered statistically significant. For the behavioral studies and immunohistochemical and neuropathological assessments ANOVA was performed included the factors of genotype, age, and treatment as warranted. All post hoc analyses used the HSD Tukey test, with *p* values<0.05 considered to be statistically significant. In the text, group means are presented ± the standard error of the mean. Details of the statistical analyses are presented in the figure legends.
